# Supplementary material for: Getting to a feasible income equality
Source: PLoS One. 2021 Mar 30;16(3):e0249204. doi: 10.1371/journal.pone.0249204 (PMC8009425; doi:10.1371/journal.pone.0249204)
Supplement: S1 Fig — The social welfare plots are normalized to the number of individual households in each subgroup (0.2N, where N is the total number of individual households in China). The total social welfare functions of China from 1990 to 2016 are maximized at βChina*=0.016∼0.019. (DOCX) [file pone.0249204.s001.docx]

**
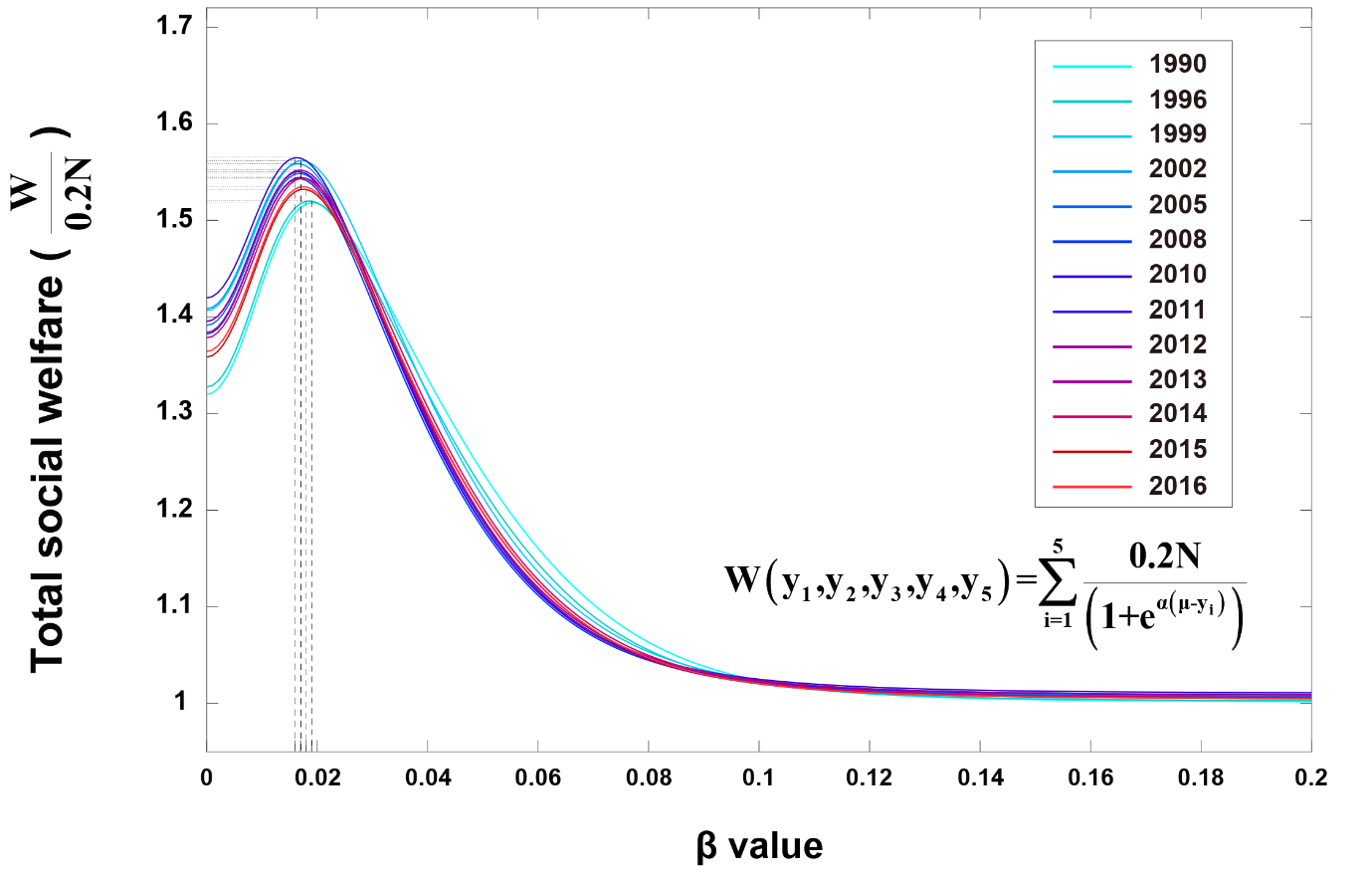
**

**S1 Fig. Total social welfare as a function of *β* value in China from 1990 to 2016.**

The social welfare plots are normalized to the number of individual households in each subgroup (0.2**N**, where **N** is the total number of individual households in China). The total social welfare functions of China from 1990 to 2016 are maximized at $\beta_{China}^{*}=0.016\sim0.019$.
