# Supplementary material for: Getting to a feasible income equality
Source: PLoS One. 2021 Mar 30;16(3):e0249204. doi: 10.1371/journal.pone.0249204 (PMC8009425; doi:10.1371/journal.pone.0249204)
Supplement: S1 Table — (DOCX) [file pone.0249204.s002.docx]

**S1 Table. Share of household income in China from 1990 to 2016.**

| **Year** | **Lowest**  **quintile** | **Second**  **quintile** | **Third**  **quintile** | **Fourth**  **quintile** | **Highest**  **quintile** |
| --- | --- | --- | --- | --- | --- |
| **1990** | 8.3 | 12.4 | 16.4 | 22.1 | 40.8 |
| **1996** | 7.4 | 11.5 | 15.9 | 22.4 | 42.9 |
| **1999** | 6.5 | 10.5 | 15.1 | 22.3 | 45.5 |
| **2002** | 5.6 | 9.6 | 14.5 | 22.3 | 48 |
| **2005** | 5.8 | 10.1 | 14.7 | 22.2 | 47.1 |
| **2008** | 5.2 | 9.4 | 14.5 | 22.5 | 48.4 |
| **2010** | 5.1 | 9.2 | 14.3 | 22.3 | 49 |
| **2011** | 5.4 | 9.6 | 14.6 | 22.3 | 48.1 |
| **2012** | 5.3 | 9.7 | 14.7 | 22.4 | 47.8 |
| **2013** | 6.2 | 10.3 | 15 | 22.1 | 46.3 |
| **2014** | 6.2 | 10.5 | 15.2 | 22.3 | 45.8 |
| **2015** | 6.4 | 10.6 | 15.3 | 22.3 | 45.4 |
| **2016** | 6.5 | 10.7 | 15.3 | 22.2 | 45.3 |

Source: World Development Indicators

https://data.worldbank.org/indicator/SI.DST.04TH.20
