# Supplementary material for: Getting to a feasible income equality
Source: PLoS One. 2021 Mar 30;16(3):e0249204. doi: 10.1371/journal.pone.0249204 (PMC8009425; doi:10.1371/journal.pone.0249204)
Supplement: S2 Table — (DOCX) [file pone.0249204.s003.docx]

**S2 Table. Parameter values for the annual social welfare functions in China from 1990 to 2016.**

| **Year** | **Critical low-income value** | **Critical high-income value** | **μ** | **α** |
| --- | --- | --- | --- | --- |
|  | $\text{L}=\frac{\left(\text{2nd Q + 3rd Q}\right)}{2}$ | $\text{H}=\frac{\left(\text{4th Q + Highest Q}\right)}{2}$ | $\text{μ = }\frac{\left(\text{L+H}\right)}{2}$ | $\text{α = }\frac{6}{\left(\text{H-L}\right)}$ |
| **1990** | 14.40 | 31.45 | 22.93 | 0.35 |
| **1996** | 13.70 | 32.65 | 23.18 | 0.32 |
| **1999** | 12.80 | 33.90 | 23.35 | 0.28 |
| **2002** | 12.05 | 35.15 | 23.60 | 0.26 |
| **2005** | 12.40 | 34.65 | 23.53 | 0.27 |
| **2008** | 11.95 | 35.45 | 23.70 | 0.26 |
| **2010** | 11.75 | 35.65 | 23.70 | 0.25 |
| **2011** | 12.10 | 35.20 | 23.65 | 0.26 |
| **2012** | 12.20 | 35.10 | 23.65 | 0.26 |
| **2013** | 12.65 | 34.20 | 23.43 | 0.28 |
| **2014** | 12.85 | 34.05 | 23.45 | 0.28 |
| **2015** | 12.95 | 33.85 | 23.40 | 0.29 |
| **2016** | 13.00 | 33.75 | 23.38 | 0.29 |
