# Supplementary material for: Getting to a feasible income equality
Source: PLoS One. 2021 Mar 30;16(3):e0249204. doi: 10.1371/journal.pone.0249204 (PMC8009425; doi:10.1371/journal.pone.0249204)
Supplement: S3 Table — (DOCX) [file pone.0249204.s004.docx]

**S3 Table. Actual and optimal income distributions in China from 1990 to 2016.**

| **Year** | **Income** | **Lowest** | **Second** | **Third** | **Fourth** | **Highest** | **Gini** |
| --- | --- | --- | --- | --- | --- | --- | --- |
|  |  | **quintile** | **quintile** | **quintile** | **quintile** | **quintile** | **coefficient** |
|  | Actual* | 8.30 | 12.40 | 16.40 | 22.10 | 40.80 | 0.30 |
| **1990** | Optimal (β*=0.019) | 15.62 | 16.89 | 18.22 | 20.30 | 28.97 | 0.12 |
|  | Difference | -7.32 | -4.49 | -1.82 | 1.80 | 11.83 | 0.18 |
|  | Actual | 7.40 | 11.50 | 15.90 | 22.40 | 42.90 | 0.33 |
| **1996** | Optimal (β*=0.019) | 15.27 | 16.51 | 17.95 | 20.30 | 29.97 | 0.13 |
|  | Difference | -7.87 | -5.01 | -2.05 | 2.10 | 12.93 | 0.19 |
|  | Actual | 6.50 | 10.50 | 15.10 | 22.30 | 45.50 | 0.36 |
| **1999** | Optimal (β*=0.017) | 15.44 | 16.53 | 17.87 | 20.20 | 29.96 | 0.13 |
|  | Difference | -8.94 | -6.03 | -2.77 | 2.10 | 15.54 | 0.23 |
|  | Actual | 5.60 | 9.60 | 14.50 | 22.30 | 48.00 | 0.39 |
| **2002** | Optimal (β*=0.017) | 15.11 | 16.17 | 17.58 | 20.07 | 31.07 | 0.14 |
|  | Difference | -9.51 | -6.57 | -3.08 | 2.23 | 16.93 | 0.25 |
|  | Actual | 5.80 | 10.10 | 14.70 | 22.20 | 47.10 | 0.38 |
| **2005** | Optimal (β*=0.017) | 15.20 | 16.35 | 17.68 | 20.09 | 30.67 | 0.14 |
|  | Difference | -9.40 | -6.25 | -2.98 | 2.11 | 16.43 | 0.24 |
|  | Actual | 5.20 | 9.40 | 14.50 | 22.50 | 48.40 | 0.40 |
| **2008** | Optimal (β*=0.017) | 14.99 | 16.10 | 17.56 | 20.11 | 31.24 | 0.15 |
|  | Difference | -9.79 | -6.70 | -3.06 | 2.39 | 17.16 | 0.25 |
|  | Actual | 5.10 | 9.20 | 14.30 | 22.30 | 49.00 | 0.40 |
| **2010** | Optimal (β*=0.016) | 15.24 | 16.27 | 17.66 | 20.07 | 30.76 | 0.14 |
|  | Difference | -10.14 | -7.07 | -3.36 | 2.23 | 18.24 | 0.27 |
|  | Actual | 5.40 | 9.60 | 14.60 | 22.30 | 48.10 | 0.39 |
| **2011** | Optimal (β*=0.017) | 15.05 | 16.17 | 17.60 | 20.06 | 31.11 | 0.14 |
|  | Difference | -9.65 | -6.57 | -3.00 | 2.24 | 16.99 | 0.25 |
|  | Actual | 5.30 | 9.70 | 14.70 | 22.40 | 47.80 | 0.39 |
| **2012** | Optimal (β*=0.017) | 15.04 | 16.21 | 17.65 | 20.12 | 30.98 | 0.14 |
|  | Difference | -9.74 | -6.51 | -2.95 | 2.28 | 16.82 | 0.25 |
|  | Actual | 6.20 | 10.30 | 15.00 | 22.10 | 46.30 | 0.37 |
| **2013** | Optimal (β*=0.017) | 15.33 | 16.44 | 17.81 | 20.09 | 30.32 | 0.13 |
|  | Difference | -9.13 | -6.14 | -2.81 | 2.01 | 15.98 | 0.23 |
|  | Actual | 6.20 | 10.50 | 15.20 | 22.30 | 45.80 | 0.36 |
| **2014** | Optimal (β*=0.017) | 15.35 | 16.51 | 17.88 | 20.18 | 30.09 | 0.13 |
|  | Difference | -9.15 | -6.01 | -2.68 | 2.12 | 15.71 | 0.23 |
|  | Actual | 6.40 | 10.60 | 15.30 | 22.30 | 45.40 | 0.36 |
| **2015** | Optimal (β*=0.018) | 15.15 | 16.34 | 17.78 | 20.17 | 30.57 | 0.14 |
|  | Difference | -8.75 | -5.74 | -2.48 | 2.13 | 14.83 | 0.22 |
|  | Actual | 6.50 | 10.70 | 15.30 | 22.20 | 45.30 | 0.36 |
| **2016** | Optimal (β*=0.017) | 15.44 | 16.59 | 17.94 | 20.17 | 29.87 | 0.13 |
|  | Difference | -8.94 | -5.89 | -2.64 | 2.03 | 15.43 | 0.23 |

Source: World Development Indicators

https://data.worldbank.org/indicator/SI.DST.04TH.20

*Actual income distribution was assumed to be the same as the share of household income.

The optimal *β* value was calculated from $\left.\frac{\partial W}{\partial\beta}\right|\begin{matrix} \\ \beta^{*} \end{matrix}=0$.

Difference = Actual income distribution – Optimal income distribution.
