# Supplementary material for: Getting to a feasible income equality
Source: PLoS One. 2021 Mar 30;16(3):e0249204. doi: 10.1371/journal.pone.0249204 (PMC8009425; doi:10.1371/journal.pone.0249204)
Supplement: Attachment — Submitted filename: Ji-Won Park_Response to Reviewers.pdf [file pone.0249204.s005.pdf]

Firstly, we thank the editor and the reviewers for the careful reading of our manuscript and the positive and constructive comments. Below, the reviewers' comments are addressed, with our point-by-point responses in bold.

Responses to Reviewer #1:

The subject of the paper is appealing. We need to have good indicators to measure income inequality because the gap between rich and poor has been unprecedentedly widening. The authors point out that the Gini coefficient has been a prevailing indicator but need an alternative measure that is realistic and feasible. I agree. I understand the intention of the argument from page 1 to page 8. However, it is getting hard to understand from page 9. They need to clarify what is "income allocation preference." Does this mean the "sum of various talents" of the individuals? If so, it sounds like justifying the idea that talented people should be affluent in proportion to their talents. (Maybe I'm wrong) The authors wrote that income should be distributed based on the income allocation preference. I am not still convinced of this argument, but I put it aside for a while, waiting for the author's reply.

We thank the reviewer for pointing out the meaning of "income allocation preference," which could be ambiguous to the readers of the manuscript.

We wanted to use the "income allocation preference" as a measure of the economic contribution that could be made from combinations of various factors such as intelligence, personalities, and physical and social skills of individuals. Based on this definition, people with higher "income allocation preference" make higher economic contributions, therefore, deserve higher income. In addition, as the reviewer pointed out correctly, people with higher talents can have higher "income allocation preference", therefore, tend to earn a higher income as well.

We do not think that the severe income inequality we are currently experiencing is simply from the positive correlation between the individual's economic contributions and income. We instead believe that the real problem in income inequality mostly originates from the fact that a few talented people earn too much income, whereas the others earn less than what they need or deserve. In this manuscript, we proposed an unbiased income distribution using the Boltzmann distribution as a resolution for this problem. In the optimal income distribution determined using the Boltzmann distribution, people with higher "income allocation preference" still earn more income,

but the highest quintile earns less than the actual income, and the lower quintiles earn more than the actual income (see the USA case in the figure below).

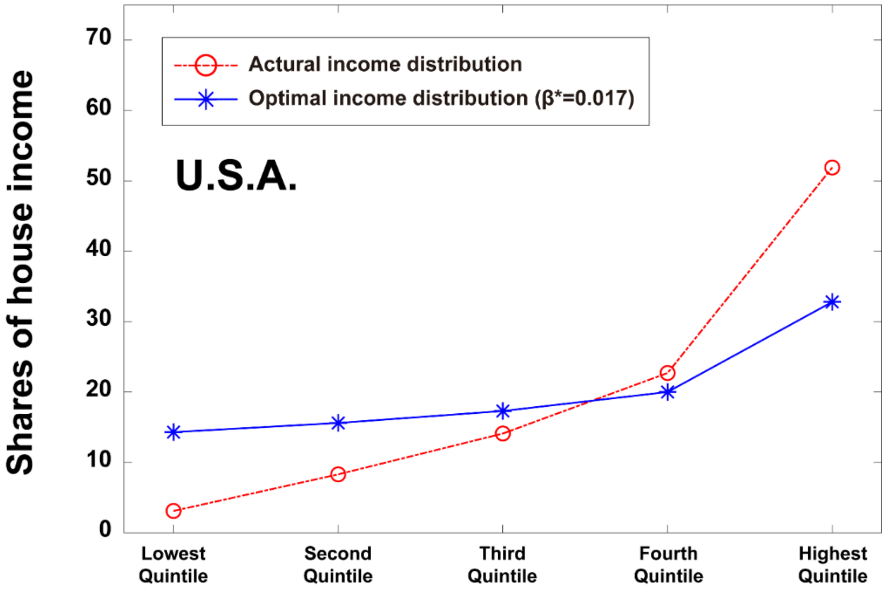

Based on the reviewer's comments, we noticed that the term "income allocation preference" itself could be quite confusing. Therefore, to help general readers grasp this critical concept more clearly, we changed the term "income allocation preference" to "*income distribution factor*" in the revised manuscript. We also modified the main text to clarify the meaning of "income distribution factor" and added the figure shown above in the revised manuscript.

Pages 9 & 11 in the revised manuscript (modified part highlighted in blue),

" The severe income inequality that we currently face is not simply due to the positive correlation between the individual's economic contributions and income. The problem instead originates from the fact that a few talented people earn too much income, whereas the others earn less than what they need or deserve. Therefore, if a given society's total income is distributed more fairly to the economic participants (individual $1, 2, \cdots, n$), the income should be distributed to the individuals in an unbiased manner ~~and purely based on the income allocation preference ($\tilde{E}_i$)~~. Such an unbiased (or fair) distribution of income can be achieved using the Boltzmann

distribution (Park et al., 2012).

……

Inspired by the fairness concept brought to the social sciences, we now apply the approach using the Boltzmann distribution in Park et al., 2012 to the income distribution. The concepts in a physical system are replaced by the concepts in an income distribution system: the physical particle is replaced by the unit income, the physical substates by the individuals in a country, and the potential energy E_i of a physical substate *i* by the negative value of the *income distribution factor* of an individual *i*. The income distribution factor ($\tilde{E}_i$) of an individual *i* is a measure of the economic contribution that could be made from combinations of various factors such as intelligence, personalities, and physical and social skills of the individual. Based on this definition, individuals with higher income distribution factors make higher economic contributions, therefore, deserve higher income. In addition, individuals with higher talents can have higher income distribution factors, therefore, tend to earn a higher income. Using the income distribution factor, the comprehensive impact of the individual's economic contributions can be quantitatively incorporated into the income distribution. "

Page 16 in the revised manuscript,

"The optimal income distributions corresponding to the social welfare maximization are summarized in Table 3. Compared to the optimal income distributions, the actual income distributions show deficiency up to the third quintile in the U.S.A, China, and Finland, and even to the fourth quintile in South Africa (Fig 5). Besides, the highest quintile in South Africa received significantly more income than the optimal income, suggesting the most biased actual income distribution (Fig 5d)."

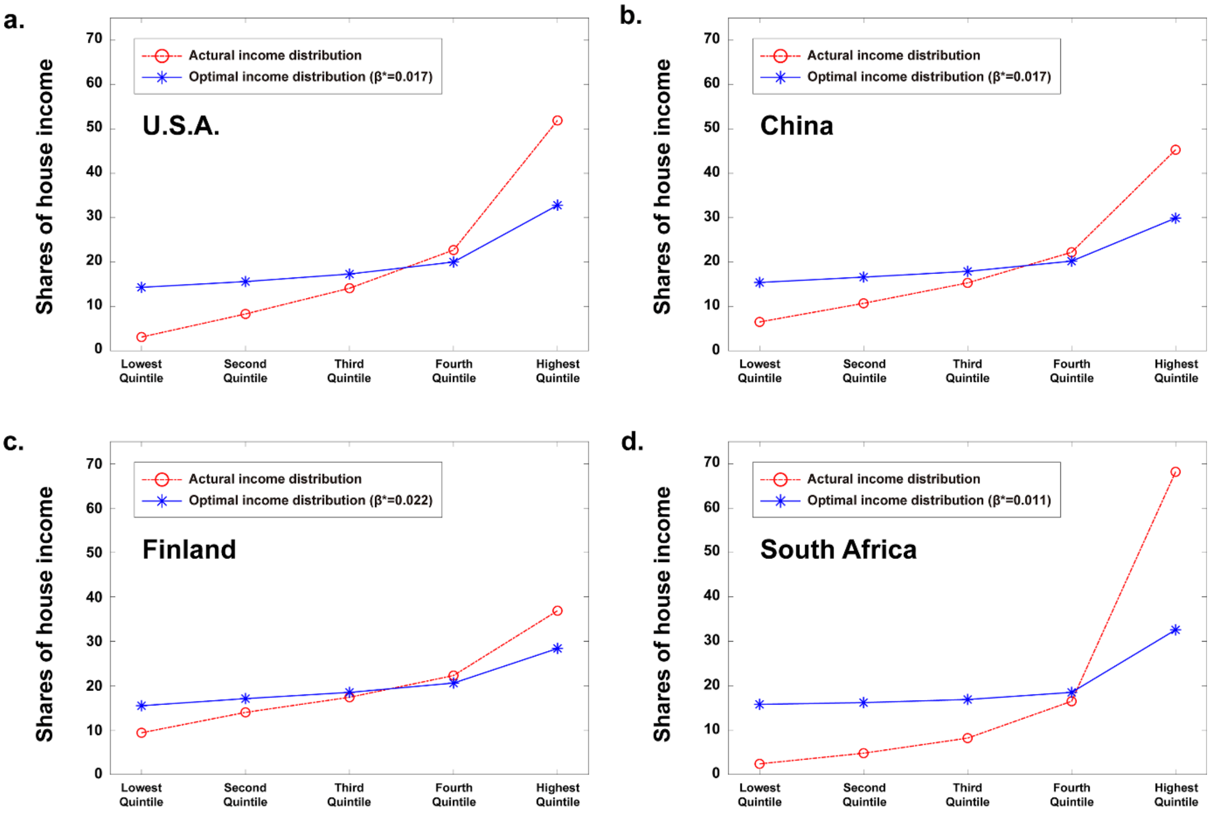

Fig 5. Actual and optimal income distributions in four countries. Compared to the actual income distribution, the optimal income distribution shows higher income in the lower quintiles and less income in the highest quintile.

The next question: why do they choose the Boltzmann distribution an appropriate measure? How does this model connect to the idea that that income should be distributed based on the income allocation preference? Although it remains some questions above, the calculation results can be an alternative indicator that we feel feasible. In conclusion, this paper is worth publishing with minor modifications.

We thank the reviewer for addressing this critical point. We want to take this opportunity to explain more explicitly how the Boltzmann distribution in physical sciences can be brought to the social sciences, such as income distribution, and how the "income distribution factor" can be connected to the Boltzmann distribution.

In physical sciences, the Boltzmann distribution yields the equilibrium probability

distribution of a physical system in its energy substates. The description is valid in a classical physics regime in which each physical particle of the system is identical to but distinguishable from the others, and the interaction among the particles is negligible. In the Boltzmann distribution, the probability (P_i) that a particle can be found in the *i*th substate is inversely proportional to the exponential function of the substate energy (E_i) (i.e., $P_i \propto e^{-\beta E_i}$, β = 1/*kT* (*k* = Boltzmann constant, *T* = absolute temperature)). The Boltzmann distribution is based on the entropy maximization and therefore provides the most probable, natural, and unbiased distribution of a physical system at thermal equilibrium. One of the best-known examples is the Maxwell-Boltzmann distribution, which describes the velocity distribution of ideal gas molecules.

The Boltzmann distribution can be brought to the social sciences by replacing the key concepts in a physical system with the key concepts in a social science system. For example, in our earlier paper (Park et al., Physica A, 2012), we brought the Boltzmann distribution to the problem of permit allocation in emissions trading to provide guidelines for allocating emissions permits among multiple countries. In the study, the physical particle was replaced by the unit emissions permit, the physical substates by the individuals of the participating countries, and the potential energy E_i of a physical substate *i* by the allocation potential energy (E_i) of an individual in country *i*. With these replacements, the probability that a unit emissions permit is allocated to a country *i* became proportional to its total population (C_i) and was inversely proportional to the exponential function of the allocation potential energy E_i (i.e. $P_i \propto C_i e^{-\beta E_i}$,). β is a positive constant

In the current manuscript, we applied a similar approach using the Boltzmann distribution to the income distribution. The concepts in a physical system were replaced by the concepts in an income distribution system: the physical particle was replaced by the unit income, the physical substates by the individuals in a country, and the potential energy E_i of a physical substate *i* by the negative value of the income distribution factor (-$\tilde{E}_i$) of an individual *i*. Then the probability that a unit income is distributed to an individual *i* becomes proportional to the exponential function of the income distribution factor $\tilde{E}_i$ (i.e. $P_i \propto e^{-\beta \tilde{E}_i}$, β is a positive constant). As in the physical sciences, we believe that the Boltzmann income distribution could

provide the most probable, natural, and unbiased distribution for the income distribution.

To help readers follow this important point more explicitly, we have added the explantions above in the revised manuscript.

Pages 9~11 in the revised manuscript (modified part highlighted in blue),

"In the physical sciences, the Boltzmann distribution yields the equilibrium probability distribution of a physical system in its energy substates (Reif, 1965, Wannier, 1987). The description is valid in a classical physics regime in which each physical particle of the system is identical to but distinguishable from the others, and the interaction among the particles is negligible. In the Boltzmann distribution, the probability (P_i) that a particle can be found in the ith substate is inversely proportional to the exponential function of the substate energy (E_i) (i.e., $P_i \propto e^{-\beta E_i}$, β = 1/kT (k = Boltzmann constant, T = absolute temperature)). The Boltzmann distribution, is based on entropy maximization and provides the most probable, natural, and unbiased distribution of a physical system at thermal equilibrium.

……

Unlike other studies, Park et al. (2012) applied entropy maximization in a different direction. Park et al. (2012) introduced entropy maximization to the problem of permit allocation in emissions trading. In their study, the concepts in a physical system were replaced by the concepts in an emissions trading system: the physical particle was replaced by the unit emissions permit, the physical substates by the individuals of the participating countries, and the potential energy E_i of a physical substate i by the allocation potential energy (E_i) of an individual in the country i. Then the probability that a unit emissions permit is allocated to a country i became proportional to its total population (C_i) and was inversely proportional to the exponential function of the allocation potential energy E_i (i.e. $P_i \propto C_i e^{-\beta E_i}$, β is a positive constant). It was argued that the Boltzmann distribution in the initial permit allocation provides the most probable allocation among multiple countries. In addition, it was proposed that the

concept of '*most probable*' in the physical sciences might be translated into '*fair*' in social sciences, as the distribution provides a natural and undistorted allocation among participants.

Inspired by the fairness concept brought to the social sciences, we now apply the approach using the Boltzmann distribution in Park et al., 2012 to the income distribution. The concepts in a physical system are replaced by the concepts in an income distribution system: the physical particle is replaced by the unit income, the physical substates by the individuals in a country, and the potential energy E_i of a physical substate *i* by the negative value of the *income distribution factor* of an individual *i*. The income distribution factor ($\tilde{E}_i$) of an individual *i* is a measure of the economic contribution that could be made from combinations of various factors such as intelligence, personalities, and physical and social skills of the individual. Based on this definition, individuals with higher income distribution factors make higher economic contributions, therefore, deserve higher income. In addition, individuals with higher talents can have higher income distribution factors, therefore, tend to earn a higher income. Using the income distribution factor, the comprehensive impact of the individual's economic contributions can be quantitatively incorporated into the income distribution. "

REIF, F. 1965. *Statistical thermal physics*, Mcgraw-Hill Kogakusha.

WANNIER, G. H. 1987. *Statistical physics,* New York, Dover Publications.

Responses to Reviewer #2:

This is an interesting proposal to improve the Gini coefficient as a better measure of income inequality of nations. I consider that this work could be of interest to a broad community of social scientists and governmental policy makers. The authors have modeled "feasible" (or optimal) income distributions that could be implemented in the calculation of new Gini coefficients of nations. They have used the sigmoid welfare function and the Boltzmann income distribution to generate such optimal income distributions and then used to calculate new Gini coefficients of four nations (USA, China, Finland and South Africa). As a consequence, the new Gini coefficients are now narrowly distributed.

The present proposal involves a rather technical methodology to determine the respective optimal income distributions of each country. For this reason, in order to get a better in site of the proposal, I suggest that the authors should extend their excise shown in Table 3 for one year, to a five-year window. In this way it will be easier to understand the evolution of the difference between the calculation of the two versions of each Gini coefficient. In particular, it will be interesting to learn how China's Gini coefficient has evolved in the last five years.

We thank the reviewer for the constructive suggestion. We agree that the time evolution of Gini coefficients provides new insight and thus should be added to the revised manuscript.

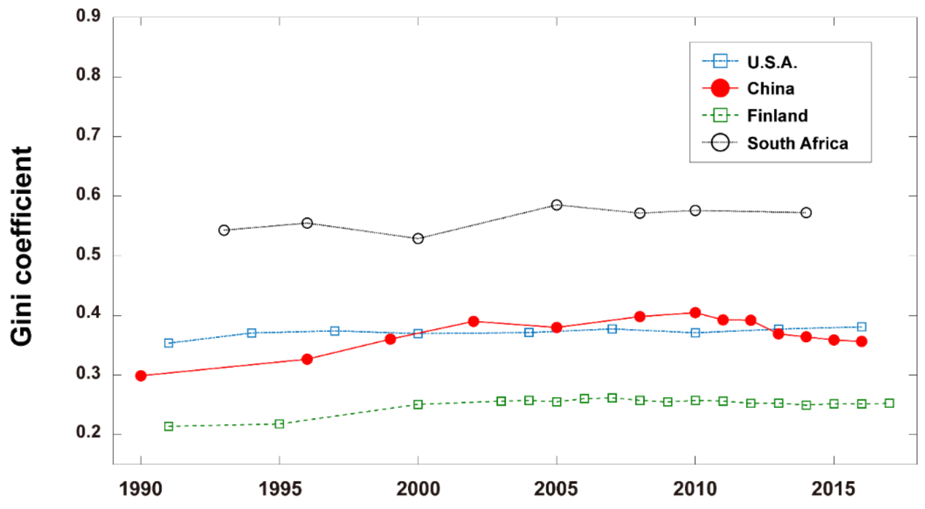

To investigate the evolution of the Gini coefficients, we first checked the Gini coefficients for the actual household income distributions (World Bank data) from early 1990 to 2016 in the four countries, the USA, China, Finland, and South Africa. As shown in the figure above, the USA and Finland show only little to small changes, and South Africa shows only sparse data points with a fluctuation over time. On the other hand, China presents noticeable changes with trackable trends. Therefore, as a representative case (also as the reviewer suggested), we have focused on the evolution of Gini coefficients in China.

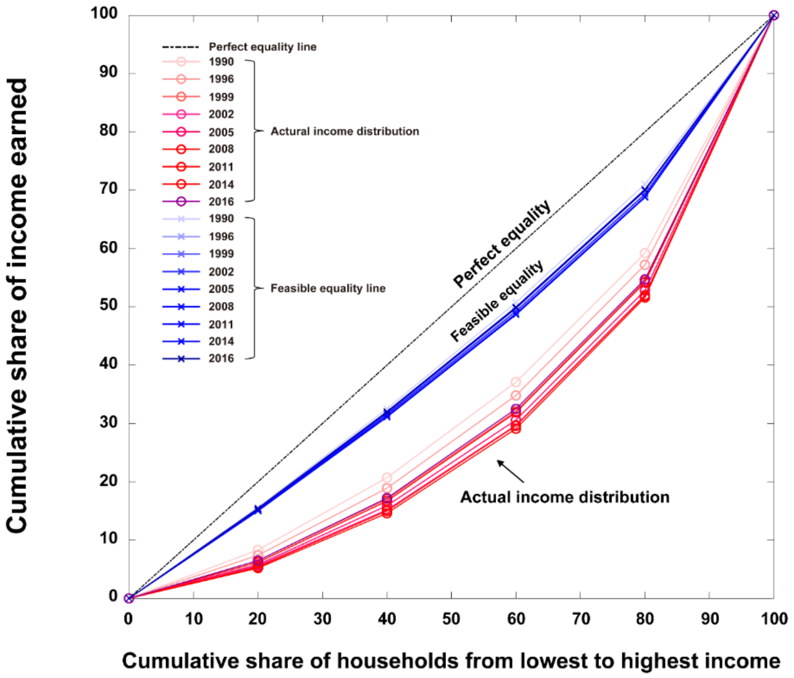

The figure above shows the Lorenz curves for the actual and the optimal income distributions in China from 1990 to 2016. The Lorenz curves for the actual income distributions are widely dispersed, whereas the Lorenz curves for the optimal income distributions, or the feasible income equality lines, are much narrowly dispersed in between the diagonal (idealistic perfect equal) and the actual income lines. From the Lorenz curves, the Gini coefficients were calculated, and the evolution of the Gini coefficients is plotted (Figure below). The Gini coefficient for the actual income distribution increases from 0.30 (1990) to 0.40 (2010), and then decreases slightly down to 0.36 (2016). On the other hand, the Gini coefficient for the optimal income distributions presents almost a flat line with little variations between 0.12 (1990) and 0.15 (2016). This result suggests that the feasible equality line could be not only universally applicable to multiple countries but also could be time-independent.

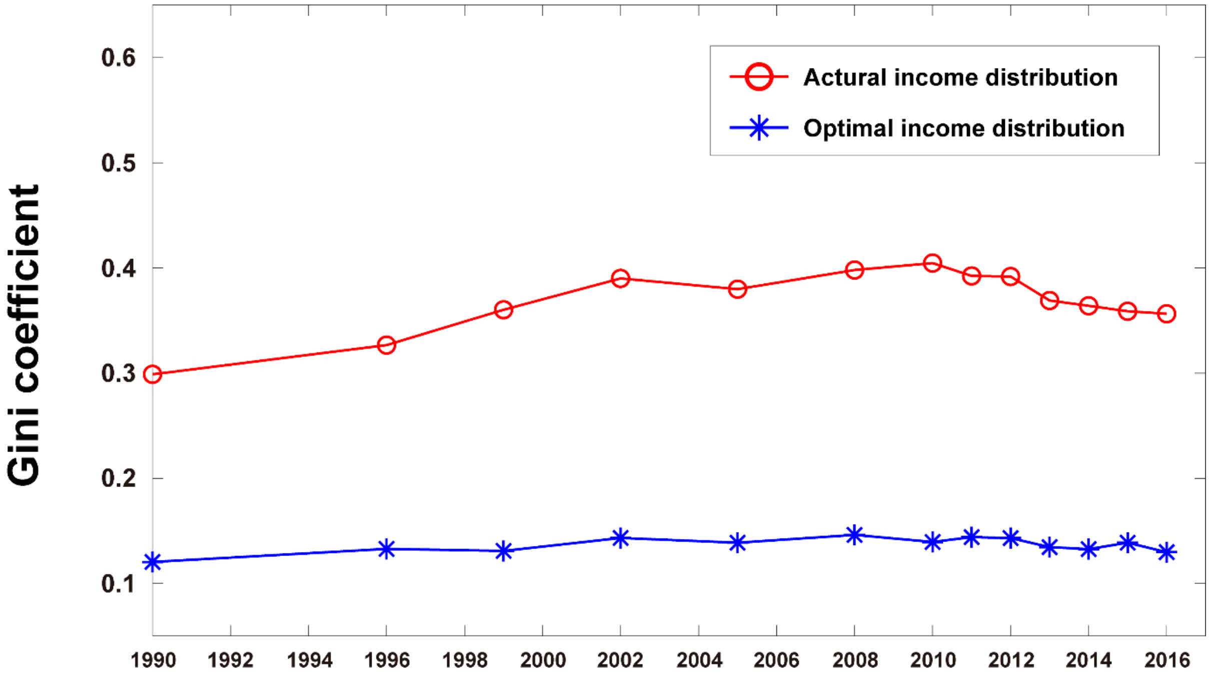

In the revised main manuscript and the supporting information, we have added detailed descriptions of the time evolution study in China from 1990 to 2016.

Pages 16~17 in the revised manuscript (modified part highlighted in blue),

"The universal feasible equality line was further investigated in the evolution of the household income dispersions in China from 1990 to 2016 (Table S1). From the income dispersions, the L, H, μ, and α values in the sigmoid individual welfare functions were calculated (Table S2). Then, the optimal income distributions were evaluated by determining the social-welfare-maximizing β values in the Boltzmann income distribution (Table S3 and Fig S1).

Fig 7 shows the Lorenz curves for the actual and the optimal income distributions in China from 1990 to 2016. The Lorenz curves for the actual income distributions are widely dispersed, whereas the Lorenz curves for the optimal income distributions, or the feasible income equality lines, are much narrowly dispersed in between the diagonal (idealistic perfect equal) and the actual income lines.

From the Lorenz curves, the Gini coefficients were calculated (Table S3), and the evolution of the Gini coefficients is plotted in Fig 8. The Gini coefficient for the actual

income distributions shows noticeable changes with trackable trends: it increases from 0.30 (1990) to 0.40 (2010), and then decreases slightly down to 0.36 (2016). On the other hand, the Gini coefficient for the optimal income distributions presents almost a flat line with little variations between 0.12 (1990) and 0.15 (2016). This result suggests that a universal feasible equality line could be time-independent, therefore serving as a reference over a long period of time. ”

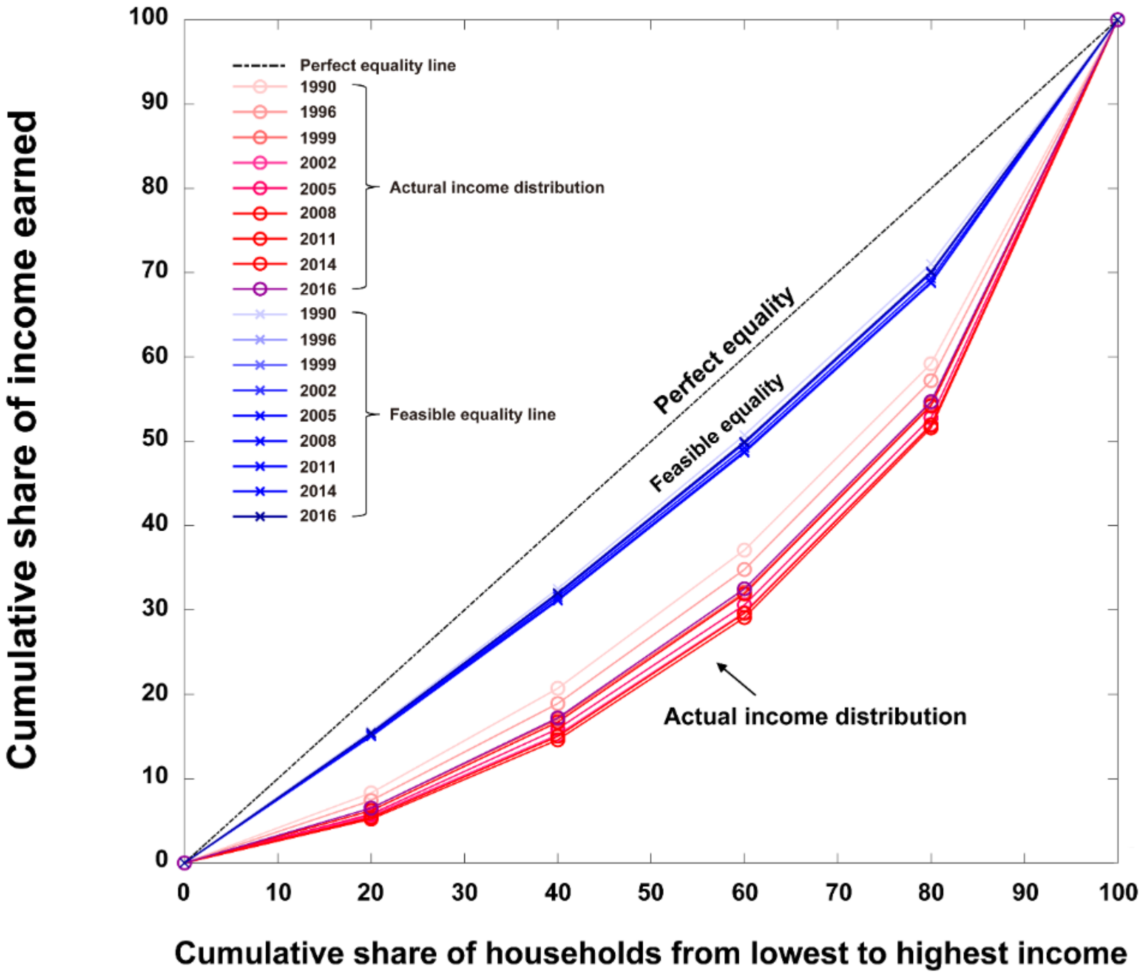

Fig 7. Lorenz curves for the actual and the optimal income distributions in China from 1990 to 2016. The corresponding Gini coefficients can be found in Table S3. Note that the feasible equality lines (blue) are similar, while the actual income distributions are widely dispersed over time.

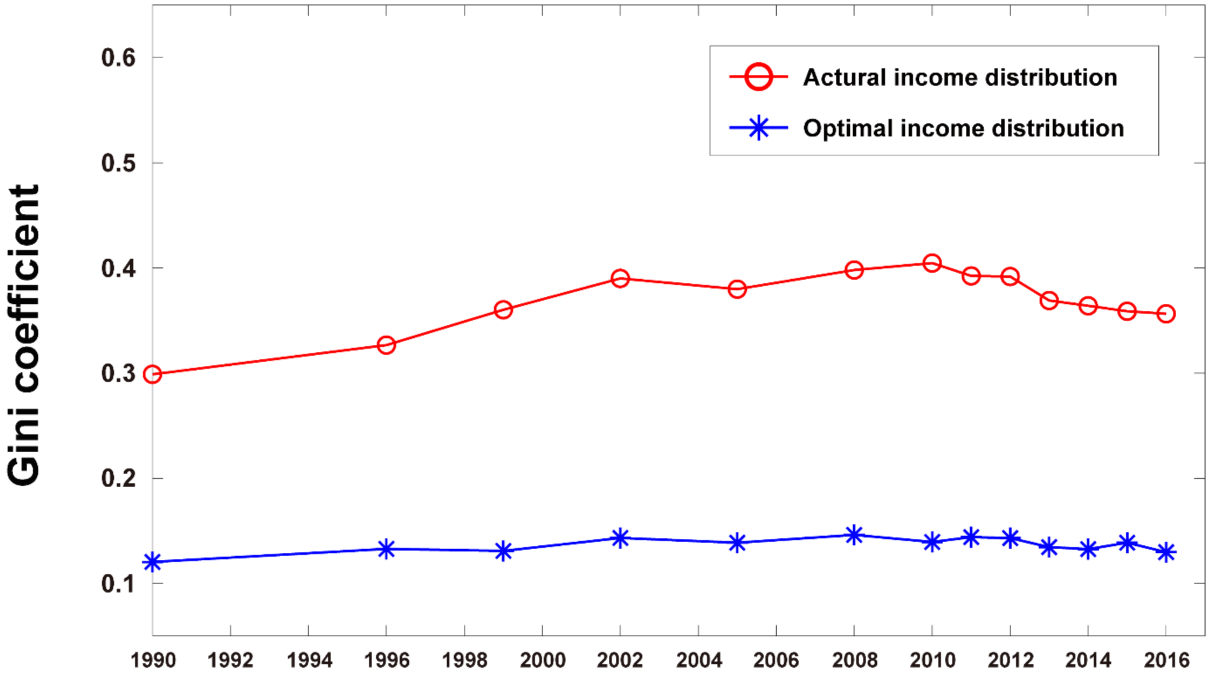

Fig 8. Evolution of Gini coefficients in China from 1990 to 2016. The Gini coefficient values at the data points can be found in Table S3.

In the supporting information,

(contined in the next pages)

Table S1. Share of household income in China from 1990 to 2016.

Year	Lowest quintile	Second quintile	Third quintile	Fourth quintile	Highest quintile
**1990**	8.3	12.4	16.4	22.1	40.8
**1996**	7.4	11.5	15.9	22.4	42.9
**1999**	6.5	10.5	15.1	22.3	45.5
**2002**	5.6	9.6	14.5	22.3	48
**2005**	5.8	10.1	14.7	22.2	47.1
**2008**	5.2	9.4	14.5	22.5	48.4
**2010**	5.1	9.2	14.3	22.3	49
**2011**	5.4	9.6	14.6	22.3	48.1
**2012**	5.3	9.7	14.7	22.4	47.8
**2013**	6.2	10.3	15	22.1	46.3
**2014**	6.2	10.5	15.2	22.3	45.8
**2015**	6.4	10.6	15.3	22.3	45.4
**2016**	6.5	10.7	15.3	22.2	45.3

Source: World Development Indicators
https://data.worldbank.org/indicator/SI.DST.04TH.20

Table S2. Parameter values for the annual social welfare functions in China from 1990 to 2016.

Year	Critical low-income value	Critical high-income value	μ	α
	$L = \frac{(2nd\ Q + 3rd\ Q)}{2}$	$H = \frac{(4th\ Q + Highest\ Q)}{2}$	$\mu = \frac{(L+H)}{2}$	$\alpha = \frac{6}{(H-L)}$
**1990**	14.40	31.45	22.93	0.35
**1996**	13.70	32.65	23.18	0.32
**1999**	12.80	33.90	23.35	0.28
**2002**	12.05	35.15	23.60	0.26
**2005**	12.40	34.65	23.53	0.27
**2008**	11.95	35.45	23.70	0.26
**2010**	11.75	35.65	23.70	0.25
**2011**	12.10	35.20	23.65	0.26
**2012**	12.20	35.10	23.65	0.26
**2013**	12.65	34.20	23.43	0.28
**2014**	12.85	34.05	23.45	0.28
**2015**	12.95	33.85	23.40	0.29
**2016**	13.00	33.75	23.38	0.29

Table S3. Actual and optimal income distributions in China from 1990 to 2016.

Year	Income	Lowest quintile	Second quintile	Third quintile	Fourth quintile	Highest quintile	Gini coefficient
1990	Actual*	8.30	12.40	16.40	22.10	40.80	0.30
	Optimal (β*=0.019)	15.62	16.89	18.22	20.30	28.97	0.12
	Difference	-7.32	-4.49	-1.82	1.80	11.83	0.18
1996	Actual	7.40	11.50	15.90	22.40	42.90	0.33
	Optimal (β*=0.019)	15.27	16.51	17.95	20.30	29.97	0.13
	Difference	-7.87	-5.01	-2.05	2.10	12.93	0.19
1999	Actual	6.50	10.50	15.10	22.30	45.50	0.36
	Optimal (β*=0.017)	15.44	16.53	17.87	20.20	29.96	0.13
	Difference	-8.94	-6.03	-2.77	2.10	15.54	0.23
2002	Actual	5.60	9.60	14.50	22.30	48.00	0.39
	Optimal (β*=0.017)	15.11	16.17	17.58	20.07	31.07	0.14
	Difference	-9.51	-6.57	-3.08	2.23	16.93	0.25
2005	Actual	5.80	10.10	14.70	22.20	47.10	0.38
	Optimal (β*=0.017)	15.20	16.35	17.68	20.09	30.67	0.14
	Difference	-9.40	-6.25	-2.98	2.11	16.43	0.24
2008	Actual	5.20	9.40	14.50	22.50	48.40	0.40
	Optimal (β*=0.017)	14.99	16.10	17.56	20.11	31.24	0.15
	Difference	-9.79	-6.70	-3.06	2.39	17.16	0.25
2010	Actual	5.10	9.20	14.30	22.30	49.00	0.40
	Optimal (β*=0.016)	15.24	16.27	17.66	20.07	30.76	0.14
	Difference	-10.14	-7.07	-3.36	2.23	18.24	0.27
2011	Actual	5.40	9.60	14.60	22.30	48.10	0.39
	Optimal (β*=0.017)	15.05	16.17	17.60	20.06	31.11	0.14
	Difference	-9.65	-6.57	-3.00	2.24	16.99	0.25
2012	Actual	5.30	9.70	14.70	22.40	47.80	0.39
	Optimal (β*=0.017)	15.04	16.21	17.65	20.12	30.98	0.14
	Difference	-9.74	-6.51	-2.95	2.28	16.82	0.25
2013	Actual	6.20	10.30	15.00	22.10	46.30	0.37
	Optimal (β*=0.017)	15.33	16.44	17.81	20.09	30.32	0.13
	Difference	-9.13	-6.14	-2.81	2.01	15.98	0.23
2014	Actual	6.20	10.50	15.20	22.30	45.80	0.36
	Optimal (β*=0.017)	15.35	16.51	17.88	20.18	30.09	0.13
	Difference	-9.15	-6.01	-2.68	2.12	15.71	0.23
2015	Actual	6.40	10.60	15.30	22.30	45.40	0.36
	Optimal (β*=0.018)	15.15	16.34	17.78	20.17	30.57	0.14
	Difference	-8.75	-5.74	-2.48	2.13	14.83	0.22
2016	Actual	6.50	10.70	15.30	22.20	45.30	0.36
	Optimal (β*=0.017)	15.44	16.59	17.94	20.17	29.87	0.13
	Difference	-8.94	-5.89	-2.64	2.03	15.43	0.23

Source: World Development Indicators
https://data.worldbank.org/indicator/SI.DST.04TH.20
*Actual income distribution was assumed to be the same as the share of household income.
The optimal β value was calculated from $\left.\frac{\partial W}{\partial \beta}\right|_{\beta^*} = 0$.
Difference = Actual income distribution – Optimal income distribution.

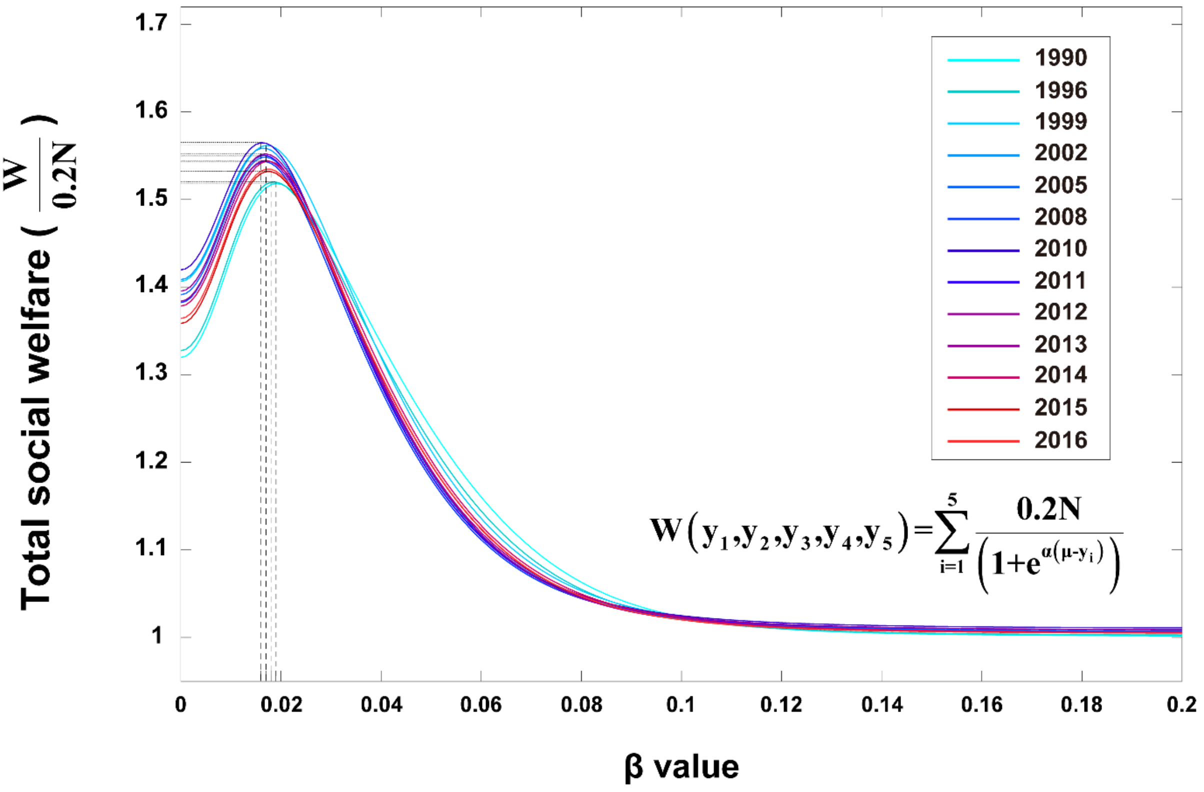

Fig S1. Total social welfare as a function of β value in China from 1990 to 2016. The social welfare plots are normalized to the number of individual households in each subgroup (0.2**N**, where **N** is the total number of individual households in China). The total social welfare functions of China from 1990 to 2016 are maximized at $\beta^{*}_{China} = 0.016 \sim 0.019$.
